# A Water-Area Recognition Approach Based on “Tuned” Texture Mask and Cuckoo Search Algorithm

**DOI:** 10.1155/2018/7690435

**Published:** 2018-12-09

**Authors:** Zhongwei Chen, Kangbo Peng, Lai Huang, Yichao Wang, Xiaozhong Wu, Zhenfeng Xiao

**Affiliations:** ^1^State Grid Hunan Electric Power Corporation Economy Institute, Changsha 410003, China; ^2^State Grid Hunan Electric Power Company Limited, Changsha 410003, China

## Abstract

Texture feature extraction is a key topic in many applications of image analysis; a lot of techniques have been proposed to measure the characteristics of this field. Among them, texture energy extracted with a mask is a rotation and scale invariant texture descriptor. However, the tuning process is computationally intensive and easily trap into the local optimum. In the proposed approach, a “Tuned” mask is utilized to extract water and nonwater texture; the optimal “Tuned” mask is acquired by maximizing the texture energy value via a newly proposed cuckoo search (CS) algorithm. Experimental results on samples and images show that the proposed method is suitable for texture feature extraction, the recognition accuracy is higher than the genetic algorithm (GA), particle swarm optimization (PSO) and the gravitational search algorithm (GSA) optimized “Tuned” mask scheme, and the water area could be well recognized from the original image. Experimental results show that the proposed method could exhibit better performance than other methods involved in the paper in terms of optimization ability and recognition result.

## 1. Introduction

Texture [1, 2] is a core property of the appearance of objects in natural scenes and is a powerful visual cue, used by both humans and machines in describing and recognizing objects of the real world. Texture feature extraction [3, 4] is a vital topic in machine vision and image analysis, which is to identify a texture sample as one of the several possible classes with a reliable texture classifier, and plays a very important role in a wide range of applications. There are kinds of texture due to changes in orientation, scale, or other visual appearances; as a result, a lot of texture feature extraction methods, such as grey-level co-occurrence matrix (GLCM), local binary pattern (LBP), Gabor wavelet, fractal theory, run-length texture descriptor, and so on [5–10], have been proposed over the years. More importantly, texture feature is one of the most significant symbols for different objects in remote sensing image, and water area is one of the most important landscape elements. Extraction of water area with image has become the favored technique to monitor urban expansion and environment, which is significant to the regional sustainable development. Some studies have been focused on the field of water area recognition by texture feature. Mettes et al. [11] proposed a hybrid texture descriptor and local detection algorithm to detect water in videos, which was able to robustly detect a region of water with high detection accuracy. Deng et al. [12] presented a high precision object-oriented water extraction scheme based on GLCM and decomposition approach, which was able to distinguish the influence of other objects and detect the small water area. However, it expends numerous features to complete the task of texture feature extraction for some traditional techniques, which need a large amount of CPU time to extract the features, and the excessive features will decrease the recognition efficiency at the same time. Although there are some methods that only need a few of features, it is difficult to stably obtain high recognition accuracy.

In order to extract the texture feature efficiently and effectively, the texture feature extraction technique based on texture mask has drawn rather considerable interest [13]. Among them, Law's mask [14] is one of the most commonly used masks to classify the different types of texture. However, the basic form of Law's mask is stationary, which is difficult to adapt various types of texture for a fixed mask [15]. Thus, You and Cohen [16] developed an adaptive texture feature extraction method called “Tuned” mask exempted from changes in rotation and scale of the texture image, and its validity has been proved. To obtain the optimal texture mask, it utilized a search strategy of gradient estimation and random search with heuristic learning. However, it is easy to lead to the high time complexity and probably trap into the local optimum.

In essence, how to obtain the optimal “Tuned” mask is a combinatorial optimization problem which may be handled by swarm intelligence algorithms. For example, Zheng and Zheng [17] utilized the genetic algorithm (GA) to search for the optimal “Tuned” mask and produced rather good results than random search. Ye et al. [18] explained the principle and steps of “Tuned” mask with the particle swarm optimization algorithm (PSO) and illustrated how to train “Tuned” mask with the proposed method in details. Wan et al. [19] introduced a residential area recognition method based on “Tuned” mask and optimized with the gravitational search algorithm (GSA), which was able to keep a good balance on the efficiency and recognition accuracy. In all, GA, PSO, and GSA could obtain good “Tuned” mask, but the dimension of the optimization problem is relatively high and the value of each individual should be a real number in the range of wide continuous space, which may not guarantee the research ability in the solution space; it is worth trying more swarm intelligence algorithms on this topic.

Cuckoo search (CS) [20] is a newly proposed swarm intelligence algorithm with stochastic global search strategy. Nowadays, the CS algorithm has been widely used in diverse applications, e.g., Wang et al. [21] utilized the CS algorithm to solve the function optimization problem and attained the optimal solution. Fouladgar et al. [22] utilized the CS algorithm to create a precise equation for predicting the ground vibration produced by blasting operations in copper mine. Suresh et al. [23] used the CS algorithm to make contrast enhancement for satellite images. In the field of texture feature extraction, Wang et al. [24] hybridized the CS algorithm with K-means algorithm to optimize the clustering center and enhanced the accuracy and efficiency of classification. Yang et al. [25] presented a remote image classification approach by learning the attribute weight of Naive Bayes classifier through the CS algorithm, which obtained higher classification accuracy and more stable performance than other evolutionary algorithms. Further, Medjahed et al. [26] proposed a new procedure for band selection by using the binary-coded CS algorithm to optimize the objective function, which could obtain satisfactory results with regard to other relevant approaches. In the paper, how to obtain the optimal “Tuned” mask is a continuous combinatorial optimization problem, which could be solved by decimal encoding. Hence, a novel water area recognition technique is proposed using “Tuned” mask and blending of the CS algorithm.

The rest of this paper is structured as follows. The idea of the proposed approach to produce the optimal “Tuned” mask by using the CS algorithm is detailed in Section 2.  Section 3 displays the experimental results and discussion. Finally, the paper is concluded in Section 4.

## 2. The Proposed Method

### 2.1. Cuckoo Search Algorithm

Cuckoo search (CS) is a novelty evolutionary algorithm proposed by Yang and Deb in 2009. The algorithm is a search strategy model on brood parasitism of some cuckoo species by laying their eggs in the nests of other host birds. If a host bird discovers the eggs are not their own, they will either throw these alien eggs away or simply abandon its nest and build a new nest elsewhere. The better new solution will take place of the solution which is relatively worse in the nest. For simplicity, only three idealized rules are used to describe the CS algorithm as follows [27]:

- Each cuckoo lays one egg at a time and dumps it in a randomly chosen nest.
- The best nests with high quality of eggs (solutions) will carry over to the next generations.
- The number of available host nests is fixed, and a host can discover an alien egg with a probability *P*_a_ ∈ [0,1]. The host bird can either throw the egg away or abandon the nest so as to build a completely new nest in a new location.

Moreover, a mass of studies have indicated that flight behaviors of many animals and insects have the typical characteristics of the Lévy flight. For an optimization problem, the quality of a solution could simply be corresponding to the fitness value of the objective function. Other forms of fitness can be defined in a parallel way to the objective function in other evolutionary algorithms. Three rules are defined in the algorithm; first, each egg in a nest stands for a solution; second, a cuckoo egg denotes a new solution; third, all of the cuckoos are evaluated by the fitness value of the objective function to be optimized and have velocities which directly decide the cuckoos' flying; the intent is to use the new better solutions to replace the not so good solution in the nests.

In order to generate the new solutions *x*^(*t*+1)^, call the bird *i*; a Lévy flight can be defined as follows:

(1) $$\begin{array}{c} x_{i}^{\left(t+1\right)}=x_{i}^{\left(t\right)}+\alpha \;⊕\text{ Levy}\left(\lambda \right), \end{array}$$

where *α* > 0 is the step size which should be connected with the solve space. The product ⊕ means entry-wise multiplications. This entry-wise product is similar to those used in PSO, the random walk via Lévy flight is more efficient in searching the solve space, and its step length is much longer in the long run.

The Lévy flight essentially provides a random walk while the random step length is drawn from a Lévy distribution, which has an infinite variance with an infinite mean as follows:

(2) $$\begin{array}{c} \text{Levy}∼u=t^{-\lambda }\left(1<\lambda \leq 3\right). \end{array}$$

Here, the consecutive steps of a cuckoo essentially from a random walk process which obeys a power law step-length distribution with a heavy tail. However, a large proportion of the new solutions may be generated by extensive randomization whose locations may be far from the current best solution; this will make sure the algorithm will not fall into a local optimum.

### 2.2. “Tuned” Mask

In order to utilize the optimal texture mask and make an accurate recognition for different texture features, You and Cohen [16] suggested the extension of Law's scheme by abandoning the traditional masks with constants and replacing them with variables in order to improve the recognition accuracy and reliability. In the method, a single 5 × 5 mask is produced which extracts a common feature of a single texture at different rotations and scales; at the same time, it discriminates this feature from other texture features to a large extent. The new mask is called a “Tuned” or adaptive mask, and the whole process of texture feature extraction is very simple.

The key issue to apply the CS algorithm is the representation of the problem, that is, how to make a suitable mapping between the problem solution and each bird. In the paper, a search space for a mask is 25 dimensions. Each dimension with continuous values and the symmetrical mask with zero sums are utilized to avoid plenty of computation, and the whole mask could be composed by only 10 parameters [17]. Therefore, the “Tuned” mask could be defined as follows:

(3) $$\begin{array}{c} {\text{mask}}_{i}=\left[\begin{array}{ccccc} x_{i}^{1} & x_{i}^{2} & -2\left(x_{i}^{1}+x_{i}^{2}\right) & x_{i}^{2} & x_{i}^{1}\\ x_{i}^{3} & x_{i}^{4} & -2\left(x_{i}^{3}+x_{i}^{4}\right) & x_{i}^{4} & x_{i}^{3}\\ x_{i}^{5} & x_{i}^{6} & -2\left(x_{i}^{5}+x_{i}^{6}\right) & x_{i}^{6} & x_{i}^{5}\\ x_{i}^{7} & x_{i}^{8} & -2\left(x_{i}^{7}+x_{i}^{8}\right) & x_{i}^{8} & x_{i}^{7}\\ x_{i}^{9} & x_{i}^{10} & -2\left(x_{i}^{9}+x_{i}^{10}\right) & x_{i}^{10} & x_{i}^{9} \end{array}\right]. \end{array}$$

As the size of the “Tuned” mask is 5 × 5 and requires being symmetrical with zero sums, so only 10 parameters in a mask need to be encoded, the layout of parameters in the mask plays a more important role for texture image classification than its actual values. Because the decimal code can be directly used for CS algorithm, the parameters of *x*_*i*_^1^, *x*_*i*_^2^, *x*_*i*_^3^, *x*_*i*_^4^, *x*_*i*_^5^, *x*_*i*_^6^, *x*_*i*_^7^, *x*_*i*_^8^, *x*_*i*_^9^, *x*_*i*_^10^ is encoded in the range of [−50, 50] for simplicity.

The “texture energy” TE could be calculated by the variance statistic within macro-window size in the training stage, which is defined as follows:

(4) $$\begin{array}{c} \text{TE}=\frac{\sum_{w_{x}}\sum_{w_{y}}F{\left(m,n\right)}^{2}}{P^{2}\times w_{x}\times w_{y}}, \end{array}$$

(5) $$\begin{array}{c} P^{2}=\underset{i,j}{\sum }A{\left(m,n\right)}^{2}, \end{array}$$

where *F*(*m*, *n*) is the image after transformation with the optimal “Tuned” mask at the pixel point (*m*, *n*), *A*(*m*, *n*) is the coding for the “Tuned” mask, and *w*_*x*_×*w*_*y*_ is a window at pixel point (*m*, *n*) (9 × 9 is used in the paper).

### 2.3. Implementation of the Proposed Method

The proposed method is simple to implement. The main process to learn the “Tuned” mask based on CS algorithm for water area recognition and texture feature extraction is in Pseudocode 1.

According to the operational process of swarm intelligence algorithms, the computational results mainly depend on parameters setting in some extent; fine tuning of the parameters can produce a better result.  Table 1 shows the parameters used in CS algorithm.

## 3. Simulation Results and Discussion

The proposed method is implemented by the language of MATLAB 2014b on a personal computer with a 2.30 GHz CPU and 8.00 GB RAM under the Windows 8 system.

As well, some existing “Tuned” mask techniques which are, respectively, proposed by Zheng and Zheng (GA [17]), Ye et al. (PSO [18]), and Wan et al. (GSA [19]) are used to make a comparison. The whole experiment is split into two parts: (1) Experiments on samples: obtain the optimal “Tuned” mask based on training samples and make recognition for water and nonwater testing samples. (2) Experiments on remote sensing images: make recognition for water areas on each pixel of the whole images. The parameters using in GA, PSO, and GSA have been shown in Tables 2–4.

To make a fair comparison, the number of function evaluations is used as terminal criterion; that is, all algorithms will stop when the number of function evaluations reaches 1000. Some contrastive experimental results are presented, including illustrative examples and performance evaluating tables, which clearly demonstrate the merits of the proposed method.

### 3.1. Experiments on Training Samples

**Figure 1:**
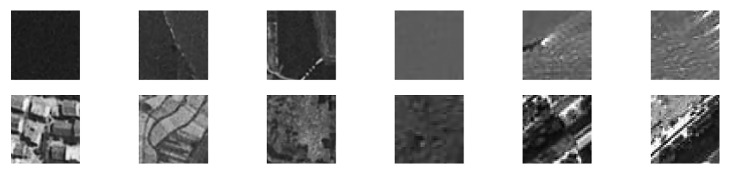
Part of training samples.

In this section, there are, respectively, 10 water and nonwater samples used for training; at the same time, 30 water and nonwater samples are utilized for testing; all of the training and testing samples are extracted from the original images. Part of training samples has been presented in Figure 1; the first row is water samples, and the second row is nonwater samples. The texture energy value of each training samples by the proposed method is listed out in Table 5, and the recognition accuracy and fitness value based on the distance between water and nonwater texture for testing samples with the optimal “Tuned” mask by using different algorithms is given in Table 6.

According to Table 5, it is discovered that the average texture energy values of water samples are 399.6 and 415.7 for two images; at the same time, the average texture energy values of nonwater samples have reached 1200; in addition, the minimum and maximum texture energy values of these two kinds of texture are significantly different, which imply that these two kinds of texture samples could be differentiated with the texture energy value. Regarding to the data in Table 6, it is clear that the CS algorithm could obtain better results than other algorithms; the fitness value is higher than “Tuned” mask techniques optimized by GA and PSO. Although the fitness value by using GSA is also well, it is still 0.6 lower than the CS algorithm. In addition, its average recognition accuracy has reached 90% for both water and nonwater samples; particularly, the CS algorithm could obtain the recognition accuracy of 100% for I2 images of nonwater samples, and the water and nonwater are accurately identified, which is a robust, reliable, and efficient method for texture feature extraction based on water areas.

### 3.2. Experiments on Remote Sensing Images

**Figure 2:**
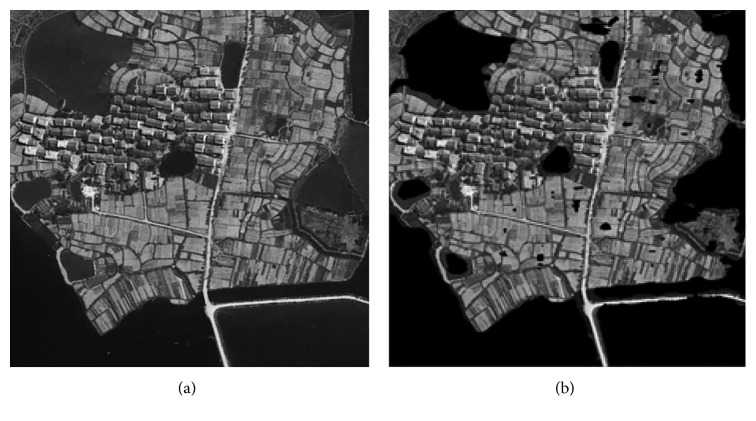
Recognition results of image 1.

**Figure 3:**
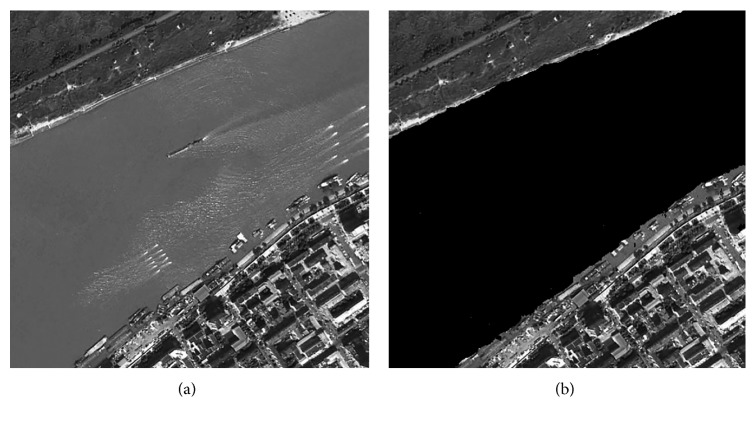
Recognition results of image 2.

After learning the optimal “Tuned” mask by the experiment on training and testing samples, 2 remote sensing images are utilized in this part and make recognition for the water areas of the whole images. The recognition results of two images have been shown in Figures 2 and 3; the left image is the original image, and the right is the recognized image; the water is marked as black color.

It is observed from Figures 2 and 3 that the water areas could be well detected with the texture energy value, which just extracts one feature to recognize, and the time complexity will be obviously reduced for the whole process. The edge selection is nearly coinciding with the original image, which could make water area recognition based on each pixel of the image. Moreover, compared with the ground truth map, the recognition accuracy could attain 91.7263% and 92.8705%, respectively, for 2 images, and the CPU time is only 0.28 second, which could meet the needs of practical application to some extent.

## 4. Conclusion

Texture feature extraction is a basic step for texture analysis. In the paper, the CS algorithm is employed to learn the optimal “Tuned” mask to solve the problem. The performance of CS algorithm has been tested on some water and nonwater samples. Moreover, results are compared with some other “Tuned” mask-based texture feature extraction techniques. The experimental results indicate that CS algorithm outperforms GA, PSO, and GSA, which has better optimization ability and could produce better “Tuned” mask. Further, the optimal “Tuned” mask is employed to detect water areas with the texture energy value and is able to obtain satisfactory recognition accuracy for practical application. In sum, “Tuned” mask has a stable performance for texture feature extraction in most cases. Further, CS algorithm could stable converge to the optimal solution by its power law step-length distribution with a heavy tail, which makes it more suitable for some practical applications.

## Figures and Tables

**Algorithm 1:**
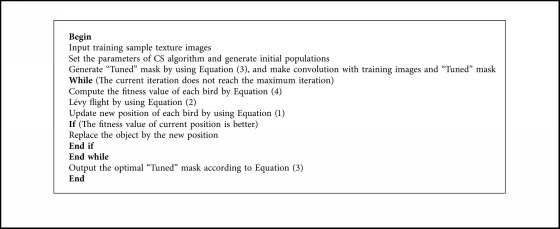
Pseudocode of learning “Tuned” mask based on CS algorithm.

**Table 1:** Parameters used in CS algorithm.

Parameter	Explanation	Value
*N*	Number of birds	20
*P*_a_	Detecting probability	0.25
*α*	Random number	[0, 1]

**Table 2:** Parameters used in GA.

Parameter	Explanation	Value
*N*	Number of genetics	20
*P*_s_	Selection ratio	0.9
*P*_c_	Crossover ratio	0.8
*P*_m_	Mutation ratio	0.01

**Table 3:** Parameters used in PSO.

Parameter	Explanation	Value
*N*	Number of particles	20
*c*_1_, *c*_2_	Positive acceleration constants	2.0
*r*_1_, *r*_2_	Random numbers	[0, 1]

**Table 4:** Parameters used in GSA.

Parameter	Explanation	Value
*N*	Number of agents	20
*G*_0_	Initial value of gravitational variable	100
*α*	User-specified constant	10

**Table 5:** Texture energies of training samples.

Image 1				Image 2			
Water samples		Nonwater samples		Water samples		Nonwater samples	
Number	TE	Number	TE	Number	TE	Number	TE
1	237.6	1	1667.5	1	387.6	1	1358.4
2	595.4	2	**936.2**	2	445.1	2	1301.8
3	478.2	3	1280.3	3	588.0	3	1222.9
4	303.3	4	1392.4	4	**670.5**	4	1187.4
5	317.8	5	1285.5	5	285.3	5	1267.0
6	277.9	6	1199.6	6	276.9	6	**1005.8**
7	283.4	7	1382.0	7	308.8	7	1142.4
8	341.2	8	1328.2	8	402.1	8	1256.2
9	457.0	9	1266.9	9	493.8	9	1325.1
10	**704.1**	10	1007.8	10	299.2	10	1287.4
Average	399.6	Average	1274.6	Average	415.7	Average	1235.4

**Table 6:** Recognition accuracies of different algorithms.

Image 1				Image 2			
Algorithm	Fitness	Recognition accuracy (%)		Algorithm	Fitness	Recognition accuracy (%)	
		Water	Nonwater			Water	Nonwater
GA	44.9455	80.0000	93.3333	GA	35.8884	86.6667	93.3333
PSO	46.5229	86.6667	93.3333	PSO	36.9465	90.0000	93.3333
GSA	47.2252	90.0000	93.3333	GSA	37.7229	93.3333	93.3333
CS	48.0444	90.0000	96.6667	CS	38.3448	93.3333	100.0000

## Data Availability

The data used to support the findings of this study are available from the corresponding author upon request.
